# Cognitive functions of the posterior parietal cortex

**DOI:** 10.3389/fnint.2013.00035

**Published:** 2013-05-09

**Authors:** Christos Constantinidis, David J. Bucci, Michael D. Rugg

**Affiliations:** ^1^Department of Neurobiology and Anatomy, Wake Forest School of MedicineWinston-Salem, NC, USA; ^2^Psychological and Brain Sciences, Dartmouth CollegeHanover, NH, USA; ^3^Center for Vital Longevity and School of Behavioral and Brain Sciences, University of Texas at DallasDallas, TX, USA

The posterior parietal cortex has traditionally been associated with visuo-spatial perception and spatial attention, however, accumulating evidence indicates that it is involved in a much wider range of cognitive functions. The articles included in the E-book review experimental data and theoretical considerations, as well as reviews of recent work supporting this idea. Anatomical, lesion, neurophysiological, and functional imaging data are discussed. Animal models (rodent and primate) as well as human studies are covered. Finally, the unique and shared functions of the posterior parietal cortex are compared to other brain areas. These contributions provide a primer of the current state of knowledge, identify unresolved questions, highlight recent conceptual and methodological advances, and, we hope, will stimulate future research.

In the first part of the E-book, evidence from rodent model systems is presented. Articles examine the contribution of animal models to long-term memory (Myskiw and Izquierdo, 2012), illusory conjunctions (Kesner, 2012), ranking of topographic signals (Broussard, 2012), and relational learning (Robinson and Bucci, 2012). A common theme across these topics is the intersection of attentional functions of posterior parietal cortex with learning/memory-related processes. Data are presented from studies that combine experimental lesion techniques and electrophysiological methods with sophisticated behavioral assays that attempt to elucidate the precise contributions of posterior parietal cortex.

A series of experiments in non-human primate models similarly reveal activation of the posterior parietal cortex in a variety of cognitive functions, such as numerosity (Roitman et al., 2012), categorization (Fitzgerald et al., 2012), and decision-making (Huk and Meister, 2012). Spatial signals are present and shape peri-personal shape and limb movements (Hadjidimitrakis et al., 2012), however, spatial information also forms an abstract spatial representation that can be decoupled from sensorimotor control (Chafee and Crowe, 2012). Neurophysiological experiments provide insights on the nature of differences between the posterior parietal cortex and other cortical areas, such as the prefrontal cortex, in the context of visual search (Wardak et al., 2012) and other tasks (Katsuki and Constantinidis, 2012). The conclusion that emerges from these studies is that the posterior parietal cortex is activated in a wide range of tasks, and individual parietal neurons exhibit neural correlates of complex cognitive functions.

In the last part of the E-book, evidence from human studies is considered. Imaging studies routinely reveal BOLD activation during episodic memory tasks (Berryhill, 2012; Levy, 2012). In recent years, nuanced memory deficits following parietal lesions have also been recognized (Berryhill, 2012). EEG and MEG studies have yielded consistent evidence about the time course of parietal mnemonic activation (Levy, 2012). Both process- and content-based models have been proposed to account for the nature of this activation (Berryhill, 2012; Levy, 2012). Finally, the posterior parietal cortex has been implicated in cognitive control, with different subdivisions proposed to be specialized for bottom-up and top-down control (Shomstein, 2012).

Collectively, these studies illustrate our current understanding of the posterior parietal cortex with regard to cognitive operations. While the nature and extent of its involvement continues to be investigated, it is now clear that its role goes beyond the functions traditionally ascribed to it, spatial representation and attention—a major development of the past few years.
